# Comparison of INTERGROWTH- 21st and Fenton growth standards to assess size at birth and at discharge in preterm infants in the United Arab Emirates

**DOI:** 10.1186/s12887-024-04928-3

**Published:** 2024-12-19

**Authors:** Leila Cheikh Ismail, Maysm N. Mohamad, Eric O. Ohuma, Mahmoud S. ElHalik, Swarup K. Dash, Tareq M. Osaili, Hayder Hasan, Mona Hashim, Sheima T. Saleh, Rameez Al Daour, Simon R. Parker, Habiba I. Ali, Lily Stojanovska, Ayesha S. Al Dhaheri

**Affiliations:** 1 Department of Clinical Nutrition and Dietetics, College of Health Sciences, University of Sharjah, Sharjah, 27272 United Arab Emirates; 2 Nuffield Department of Women’s & Reproductive Health, University of Oxford, Oxford, OX1 2JD UK; 3 Department of Nutrition and Health, College of Medicine and Health Sciences, United Arab Emirates University, Al Ain, 15551 United Arab Emirates; 4 Maternal, Adolescent, Reproductive & Child Health Centre, London School of Hygiene & Tropical Medicine, London, UK; 5 Neonatal intensive care unit, Department of Pediatrics, Latifa women and Children’s Hospital, DAHC, United Arab Emirates; 6 Department of Nutrition and Food Technology, Faculty of Agriculture, Jordan University of Science and Technology, 22110 Irbid, Jordan; 7 Institute for Health and Sport, Victoria University, Melbourne, 14428 Australia

**Keywords:** Fenton, Growth standards, INTERGROWTH-21st, Percentiles, Preterm infants

## Abstract

**Background:**

Accurate growth assessment of preterm infants is essential in guiding medical care and suitable nutritional interventions. Currently, different growth references are used across hospitals in the United Arab Emirates (UAE). This study aims to compare the INTERGROWTH-21st standards with Fenton growth references regarding birth size classification and at the time of discharge in a sample of preterm infants in the UAE.

**Methods:**

A retrospective single-center evaluation of medical records of infants born < 37 weeks of gestation was conducted using data from 2018 to 2020. Anthropometric measurements (weight, length, and head circumference) were obtained at birth and at the time of discharge, and then converted to percentiles according to the two reference standards.

**Results:**

A total of 1537 infants with a median birth gestation of 35.3 weeks, and a median birthweight of 2320 g were included. The rates of SGA, AGA, and LGA at birth were 11.5%, 80.42%, and 9.08% using INTERGROWTH-21st growth charts compared to 9.5%, 83.2%, and 7.3% respectively according to Fenton charts. The findings indicated statistically significant differences between the two growth charts classifying of preterm infants based on weight, length, and head circumference (*p* < 0.05). For every 5 cases assessed as SGA at discharge according to Fenton charts, only 3 were classified as SGA by INTERGROWTH-21st curves.

**Conclusions:**

Differences exist between the two growth charts with only moderate agreement. Thus, there is a need for harmonizing growth assessment standards. Misclassification of these vulnerable infants would affect their in-hospital and post-discharge nutrition and medical care plan.

## Introduction

Premature birth is defined as live delivery before reaching a full term of gestation (< 37 weeks), and it may entail short and long-term detrimental complications on the child’s health and wellbeing [1]. In 2020, the worldwide preterm birth rate was estimated at 9.9% of total births, accounting for 13.4 million preterm births, with rates over the past decade remaining static, and 23·4 million were small for gestational age (SGA) (one in five livebirths) [2]. A myriad of premature birth risk factors has been well established in several epidemiological studies, most of which are highly associated with maternal characteristics such as maternal age beyond 35 years and ethnicity, health and nutritional status before conception, and quality of life [3–6]. Preterm birth, is linked to both immediate and prolonged complications, including compromised health and growth, intellectual and mental disabilities, and the early onset of chronic diseases [7–9]. Additionally, it is a key contributor to the global under-5 mortality rate (U5MR) which reached 37.7 deaths per 1000 livebirths in 2019 [10]. Mortality risk is highest for preterm birth, especially at lower gestational ages. Advancements in perinatal medicine have increased the survival rates of premature infants, particularly those experiencing intrauterine and extrauterine growth restriction [11]. However, assigning the intrauterine growth status of preterm neonates at birth can be challenging. Intrauterine growth restriction (IUGR), marked by insufficient fetal growth, is crucial to identify due to its link to increased risks of perinatal mortality, morbidities, and abnormal long-term outcomes. Typically, IUGR is identified by delivering infants who are SGA, defined as having a birth weight below the 10th percentile on growth charts [12]. Hence, it is important to closely monitor the growth of preterm infants to detect any deviations from the normal trajectory. Precision in assessment is also crucial for determining appropriate nutrition and medical care [13], The method chosen for postnatal growth assessment in preterm infants significantly influences nutritional interventions and growth outcomes [14]. Furthermore, accurate postnatal growth assessment is essential for monitoring progress and enabling direct comparisons on a global scale. Multiple growth charts are utilized for this categorization.

Growth charts have been well recognized as an essential tool to assess the adequacy of received nutrition and to screen for potential deviations from normal growth that may convey adverse health conditions [15]. The Fenton growth references, which were revised and updated in 2013 after a meta-analysis of six studies (Germany, United States, Italy, Australia, Scotland, and Canada), have been widely used for the assessment of the preterm population [13, 16]. These charts incorporate recent population-based survey data including sex-specific information, aligning with WHO growth curves at 50 weeks of post-menstrual age. However, limitations include being a growth reference (describes the growth of a sample of individuals) rather than a standard (describes the growth of a ‘healthy’ population) [17], and variability in the methods of measuring growth parameters. Moreover, the usage of Fenton growth charts in monitoring postnatal growth has been associated with several drawbacks pertaining to their failure to reflect the adaptation of the premature newborn to postnatal extra-uterine life and the under-or overestimation of the newborn’s growth [18–22]. In 2015, the INTERGROWTH- 21st Project developed standards for newborn size and postnatal growth in preterm infants from a multicenter, multiethnic, population-based project conducted in eight different geographical areas (Brazil, China, Italy, Kenya, Oman, UK, and the USA) [21, 23, 24]. The methodology used was similar to the WHO child growth standard charts which are used globally and they are also sex-specific [25]. The key difference between the Fenton and INTERGROWTH- 21st preterm charts are that INTERGROWTH charts aimed to describe optimal growth that can be implemented internationally and facilitate direct comparisons across diverse settings rather than a descriptive growth reference such as the Fenton charts. Moreover, compared to Fenton growth references, INTERGROWTH- 21st preterm postnatal charts were developed specifically to monitor the extrauterine growth using actual postnatal growth data after preterm birth [20].

Hospitals in the United Arab Emirates (UAE) use different growth references for the growth assessment of infants and there are no studies to date that compare the disparities in the interpretation of these growth charts. Therefore, it is particularly important to understand differences in the assessment and interpretation between the INTERGROWTH- 21st standards and Fenton growth references as this would affect in-hospital and post-discharge nutrition plan of these vulnerable infants. As evidence shows and in line with the objectives of this study, it is hypothesized that the incidence of intrauterine and postnatal growth restriction in premature infants might change when assessed with the INTERGROWTH- 21st standards compared with the Fenton-2013 growth references. Therefore, this study aims to compare the INTERGROWTH- 21st standards with Fenton growth references regarding birth size classification and at the time of discharge in a sample of preterm infants in the UAE using data from 2018 to 2020.

## Methods

### Study participants

**Fig. 1:**
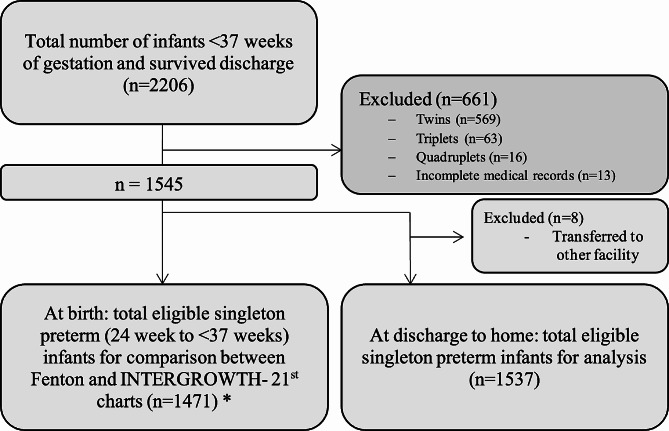
Flowchart of the study population and inclusion criteria of the study. * *n* = 74 infants were not eligible for comparison at birth as INTERGROWTH- 21st newborn size standards are not applicable for infants born < 24 weeks

A retrospective evaluation of medical records of infants born <37 weeks of gestation at Latifa Women and Children Hospital, Dubai, UAE, between July 2018 to July 2020 was conducted. Latifa Women and Children Hospital is a public hospital in Dubai that provides medical care specifically for women and children and has the largest neonatal and premature neonatal intensive care unit in the Northern Emirates. We excluded preterm infants who suffered from comorbidities that may affect normal growth such as severe malformations, genetic syndromes, and metabolic diseases, and newborns who died during hospitalization or were transferred to another hospital (*n*=100), and twins (*n*=569), triplets (*n*=63), quadruplets (16), and those who had incomplete medical records (*n*=13) (Fig. 1). A total of 2206 preterm infants were eligible for this study. Of the 2206 preterm infants, 1471 (66.7%) were eligible for analyses comparing the Fenton and INTERGROWTH- 21^st^ charts for newborn size between 24^+0^ weeks to <37 weeks (Fig. 1). At discharge to home (32^+3^ weeks to 64^+2^ weeks), 1537 (69.7%) were included in the analysis.

### Data collection

A researcher reviewed the electronic health records of preterm infants to obtain data on hospitalization, birth, and growth. Characteristics of the pregnancy (maternal age, administration of antenatal steroid, mode of delivery, and health complications during the pregnancy), anthropometric measurements of infants at birth and discharge (weight, length, and head circumference), sex, morbidities related to prematurity, length of hospitalization, and feeding practices were also recorded.

The most common method for gestational age (in weeks) assessment was the early prenatal ultrasound; if unavailable, the date of the last menstruation was used. The gestational age was used to calculate the age of the neonates at discharge by adding it to the chronological age in weeks. Anthropometric measurements (weight, length, and head circumference) were obtained for all infants at birth and at the time of discharge. At the study location, anthropometric measurements were conducted using standard techniques [26]. Weight was measured within 24 h of birth using an electronic weight scale and was recorded in grams (g) (accuracy of 10 g). An infantometer was used to measure the length in centimeters (cm) (accuracy of 0.1 cm) and a flexible, nonstretchable tape was used for the measurement of head circumference in cm (accuracy of 0.1 cm).

### Statistical analysis

Anthropometric data at birth and discharge were converted to respective centiles and Z-scores in Stata 18 according to Fenton-2013 [13, 21] and INTERGROWTH- 21st charts for newborn size and preterm postnatal growth [13, 21, 24]. We applied the INTERGROWTH-21st in Stata using the gigs module [27]. We classified anthropometric measurements of weight/length/head circumference as small for gestational age (SGA) < 10th percentile, appropriate for gestational age (AGA) between 10th and 90th percentile, and large for gestational age (LGA) > 90th percentile accordingly using the Fenton and INTERGROWTH- 21st charts.

Descriptive analyses were used to summarize maternal demographic characteristics, newborn characteristics, and outcomes, and these were recorded during hospitalization using frequencies and percentages for categorical data and mean (standard deviation, SD) or median (inter-quartile range, IQR) for continuous variables, as appropriate.

The proportions test was used to evaluate whether the percentage differences in those classified as SGA, AGA, or LGA for weight/length/head circumference differed between the Fenton and INTERGROWTH- 21st charts when compared at birth and discharge separately (using INTERGROWTH- 21st charts for newborn size were at birth and INTERGROWTH- 21st preterm postnatal growth at discharge). We also calculated the percentage of agreement between the two charts in the classification of SGA, AGA, or LGA for weight/length/head circumference at birth and at discharge.

Ethical approvals were obtained from the Research Ethics Committee at the University of Sharjah (UOS) (Reference number: REC_20_05_20_01) and Dubai Scientific Research Ethics Committee at Dubai Health Authority (Reference number: DSREC-11/2020-23). The study was conducted following the ethical standards laid down in the 1964 Helsinki Declaration. No identification information was collected to assure confidentiality and anonymity of participants. In consideration of the retrospective nature of the study, obtaining individual informed consent from subjects was not feasible. Therefore, a consent waiver was granted from the Research Ethics Committee at the University of Sharjah (UOS) (Reference number: REC_20_05_20_01) and Dubai Scientific Research Ethics Committee at Dubai Health Authority (Reference number: DSREC-11/2020-23), following thorough review of the research protocol, recognizing the minimal risk posed using anonymized and de-identified data.

## Results

### Baseline characteristics of the study population

The baseline maternal and infant demographic and clinical characteristics of the study population are summarized in Table 1. Of the 1471 infants included in the comparison between Fenton and INTERGROWTH- 21st charts at birth 53% were males, the median birth gestation was 35.4 weeks, and the median birthweight was 2370 g. The median length of stay was 5 days.

**Table 1:** Baseline characteristics of the study population

	< 37 weeks (discharge comparison)	24 to < 37 weeks (birth comparison)
Variable	Median (IQR), Frequency (Percentage) (*n* = 1545)	Median (IQR), Frequency (Percentage) (*n* = 1471)
**Maternal characteristics**		
Age (years)	32 (28–37)	33 (28–37)
C-section (%)	984 (63.7)	949 (64.6)
Gestational hypertension (%)	37 (2.4)	37 (2.5)
Gestational diabetes mellitus (%)	16 (1.0)	15 (1.0)
Antenatal steroid (%)	694 (44.9)	632 (43.0)
Premature rupture of membrane (%)	204 (13.2)	190 (12.9)
Oligohydramnios (%)	19 (1.2)	17 (1.2)
Chorioamnionitis (%)	3 (0.2)	3 (0.2)
**Characteristics of newborns**		
Sex (male, %)	817 (52.9)	773 (52.6)
Gestational age (weeks)	35.3 (32.9–36.3)	35.4 (33.6–36.4)
PMA at discharge (weeks)	36.6 (35.7–37.1)	36.6 (35.7–37.1)
Birth weight (g)	2320 (1810–2710)	2370 (1935–2725)
Weight at discharge (g)	2335 (2030–2690)	2340 (2040–2690)
Birth length (cm)	46.0 (42.0–48.0)	46.0 (43.0–48.0)
Length at discharge (cm)	46.0 (44.0–48.0)	47.0 (44.0–48.0)
Birth head circumference (cm)	32.0 (30.0–33.0)	32.0 (30.5–33.0)
Head circumference at discharge (cm)	32.5 (31.0–33.5)	32.5 (31.5–33.5)
**Hospitalization**		
Invasive mechanical ventilation (%)	577 (37.3)	513 (34.9)
Respiratory Distress Syndrome (%)	662 (42.8)	593 (40.3)
Anemia, needing blood transfusion (%)	111 (7.2)	61 (4.2)
Jaundice, needing phototherapy (%)	800 (51.8)	753 (51.2)
Patent ductus arteriosus, hemodynamically significant (%)	114 (7.4)	74 (5.0)
Sepsis, clinical or culture-proven (%)	200 (12.9)	163 (11.1)
Necrotizing enterocolitis, requiring surgical intervention (%)	13 (0.8)	9 (0.6)
Intraventricular hemorrhage (IVH), Grade 3–4 (%)	12 (0.8)	4 (0.3)
Bronchopulmonary dysplasia (%)	53 (3.4)	16 (1.1)
Exclusive breastfeeding (%)	325 (21.0)	321 (21.8)
Breast milk + fortifier (%)	1194 (77.2)	1122 (76.3)
Only formula feeding (%)	15 (1.0)	15 (1.0)
Time to regain birth weight (days)	11 (5–16)	11 (4–15)
Length of hospital stay (days)	6 (3–20)	5 (3–17)

PMA: postmenstrual age; continuous data: median (interquartile range); categorical data: counts (percentages)

Using INTERGROWTH- 21st growth charts the rates of SGA, AGA, and LGA were11.5%, 80.4%, and 8.1% compared to 9.5%, 83.2%, and 7.3% respectively according to Fenton charts (Table 2). Using length percentiles of Fenton and INTERGROWTH- 21st curves, the number of preterm infants classified below the 10th percentile at birth was 121 (8.2%) vs. 105 (12.0%) and above the 90th percentile 157 (10.7%) vs. 517 (16.9%), respectively (*p* < 0.001). For head circumference, the rates of below the 10th and above the 90th percentile at birth using INTERGROWTH- 21st growth charts were 6.8% and 17.7%% compared to 4.3% and 16.7% respectively according to Fenton charts.

Likewise, at discharge, the INTERGROWTH- 21st growth charts had lower rates of SGA (21.8%) compared to Fenton charts (35.1%), but higher rates of LGA (7.61% vs. 1.7%, respectively). Length percentiles followed a similar trend of discharge, with 21.5% classified below the 10th percentile using INTERGROWTH- 21st growth charts vs. 23.2% using Fenton charts and 17.3% classified above the 90th percentile using INTERGROWTH- 21st growth charts vs. 4.2% using Fenton charts.

**Table 2:** Classification of weight, length, and head circumference according to Fenton and INTERGROWTH- 21st postnatal growth charts at birth, and discharge

	Fenton-2013	INTERGROWTH- 21st	*P* value
**At birth (n = 1471): 27** ^+ 0^ **to < 37 weeks**			
SGA (n, %)	140 (9.5)	169 (11.5)	0.0812
AGA (n, %)	1224 (83.2)	1183 (80.42)	0.05
LGA (n, %)	107 (7.3)	119 (8.09)	0.4061
Length < 10th percentile (n, %)	121 (8.2)	176 (12.0)	< 0.001
Length 10-90th percentile (n, %)	1193 (81.1)	1047 (71.2)	< 0.001
Length > 90th percentile (n, %)	157 (10.7)	248 (16.9)	< 0.001
HC < 10th percentile (n, %)	63 (4.3)	100 (6.8)	0.0029
HC 10-90th percentile (n, %)	1163 (79.1)	1111 (75.53)	0.0221
HC > 90th percentile (n, %)	245 (16.7)	260 (17.7)	0.04633
**At discharge (n = 1537): 32** ^**+ 3**^ **weeks to 64** ^**+ 2**^ **weeks**			
SGA (n, %)	540 (35.1)	335 (21.8)	< 0.001
AGA (n, %)	971 (63.18)	1085 (70.6)	< 0.001
LGA (n, %)	26 (1.7)	117 (7.61)	< 0.001
Length < 10th percentile (n, %)	356 (23.2)	330 (21.5)	0.26
Length 10-90th percentile (n, %)	1116 (72.61)	941 (61.2)	< 0.001
Length > 90th percentile (n, %)	65 (4.2)	266 (17.3)	< 0.001
HC < 10th percentile (n, %)	271 (17.6)	350 (22.8)	< 0.001
HC 10-90th percentile (n, %)	1170 (76.21)	936 (60.9)	< 0.001
HC > 90th percentile (n, %)	96 (6.3)	252 (16.3)	< 0.001

Table 3 shows the agreement between Fenton and INTERGROWTH- 21st growth charts regarding the classification of weight, length, and head circumference at birth and discharge. The lowest agreement between the two charts was found for the classification of SGA at discharge. Fenton classified 540 infants as SGA at discharge, out of which 62.0% (*n* = 335) were also classified as SGA according to INTERGROWTH- 21st standards. This lack of agreement was not present at birth, where Fenton classified 140 infants as SGA, out of which 99.3% (*n* = 139) were also classified as SGA according to INTERGROWTH- 21st standards.

The agreement between INTERGROWTH- 21st and Fenton regarding the classification of length below the 10th percentile was 85.1% at birth and 91.6% at discharge. Whereas there was a 100% agreement between the two standards for the classification of head circumference below the 10th percentile at birth and discharge.

**Table 3:** Comparing weight, length, and head circumference classification according to INTERGROWTH- 21st and Fenton in preterm infants

Variable		*n* according to Fenton only	*n* (%) according to both Fenton and INTERGROWTH- 21st
**Weight**	**At birth (n = 1471)**	SGA, (*n* = 140)	139 (99.3)
		AGA, (*n* = 1224)	1175 (96.0)
		LGA, (*n* = 107)	100 (93.5)
	**At discharge (n = 1537)**	SGA, (*n* = 540)	335 (62.0)
		AGA, (*n* = 971)	880 (90.6)
		LGA, (*n* = 26)	26 (100)
**Length**	**At birth (n = 1471)**	< 10th percentile, (*n* = 121)	121 (85.1)
		10-90th percentile, (*n* = 1193)	1019 (69.7)
		> 90th percentile (*n* = 157)	119 (100)
	**At discharge (n = 1537)**	< 10th percentile, (*n* = 356)	326 (91.6)
		10-90th percentile, (*n* = 1116)	911 (81.6)
		> 90th percentile (*n* = 65)	65 (100)
**Head circumference**	**At birth (n = 1471)**	< 10th percentile, (*n* = 63)	63 (100)
		10-90th percentile, (*n* = 1163)	1065 (91.2)
		> 90th percentile (*n* = 245)	199 (21.2)
	**At discharge (n = 1537)**	< 10th percentile, (*n* = 271)	271 (100)
		10-90th percentile, (*n* = 1170)	936 (80.0)
		> 90th percentile (*n* = 96)	96 (100)

## Discussion

This retrospective study compared INTERGROWTH- 21st and Fenton growth standards in evaluating both growth patterns of preterm infants at birth and at discharge. The findings indicated statistically significant differences between the two growth charts in the classification of preterm infants based on weight, length, and head circumference. The results also showed that for every five cases assessed as SGA at discharge according to Fenton charts, only three were classified as SGA by INTERGROWTH- 21st curves. Accordingly, the results confirm that differences exist between the two growth charts and suggest only moderate agreement between them. Misclassification of these vulnerable infants due to differences in weight assessment using growth charts would also affect their in-hospital and post-discharge nutrition and care plan.

Available literature indicated disparities in the interpretation of growth parameters when different growth charts are used [28, 29]. Similarly, the results of the study showed that the incidence of SGA at birth and discharge was significantly lower using the INTERGROWTH- 21st growth standards compared to Fenton charts. Lebrao et al. conducted a study among 173 preterm infants (aged between 26 and 33 weeks) in Brazil and concluded that the incidence of SGA was significantly higher when using the Fenton curves compared to the INTERGROWTH- 21st growth standards [30]. Likewise, Patel et al. concluded that Fenton curves reported more intrauterine and extrauterine growth restriction as compared to the INTERGROWTH- 21st standards [31]. The latter study reported poor agreement between the two growth standards. In contrast, a study conducted in Turkey among 248 very preterm infants found that one out of every four cases assessed as SGA according to the INTERGROWTH- 21st standards were within the normal interval according to Fenton curves [22]. Similarly, Reddy et al. conducted a prospective study in India among 603 premature infants (≤ 32 weeks GA) and reported that about 3% of infants who were assessed as AGA on Fenton curve were identified as SGA by INTERGROWTH- 21st standards [32]. Differences between studies might be due to using different methods for the assessment of gestational age and anthropometric measurements, or due to differences in the studied population as in the case of the Turkish and Indian studies where they had more premature infants in their studies as compared to the current study. It is also noteworthy to highlight that discrepancies may be associated with the absence of certain ethnicities in growth standards, particularly considering that the Fenton curves were derived from developed countries and do not include data from mothers and babies of Middle Eastern or Arab descent. In contrast, the INTERGROWTH-21st included data from Oman, offering a more inclusive representation for a diverse range of populations. Moreover, INTERGROWTH- 21st curves were developed based on real postnatal growth data following preterm birth which could also influence such discrepancies.

In Lebanon, a retrospective study was conducted on 318 premature babies to compare the two growth charts (Fenton and INTERGROWTH- 21st ) reported controversial results, as Fenton was found better at predicting newborns’ growth in weight, height, and cranial perimeter before two weeks of age and at four weeks, while INTERGROWTH- 21st showed higher percentiles at two weeks for weight and length [33]. The authors of the latter study suggested the need for conducting clinical trials or prospective studies on the national level before selecting which standards to implement. Moreover, there is a need for continuous research to delve into the clinical significance of diagnosing SGA and to further improve healthcare interventions for these vulnerable babies.

The literature highlights the importance of accurate diagnosis of SGA as these infants not only experience increased rates of morbidity and mortality during the neonatal period but also face a heightened risk of health issues later in life, such as short stature, puberty disorders, metabolic syndrome, neurocognitive dysfunction and metabolic complications including insulin resistance, obesity, and hypertension later in life [34, 35]. As such, precisely identifying various categories of vulnerable newborns is essential for personalized care and advancing primary prevention efforts. This includes understanding causal mechanisms and enhancing targeted clinical interventions [36]. Moreover, newborns classified as SGA are usually supplemented with hypercaloric nutrition to compensate for growth restriction. Hence improved weight gain has been related to better neurodevelopmental outcomes in preterm infants [37]. The literature has also shown that preterm infants who are mainly breastfed have enhanced neurodevelopmental consequences regardless of having lower weight gain [38, 39]. On the other hand, studies are suggesting an association between preterm birth and obesity in adulthood [40, 41]. Therefore, it is worth exploring if “overfeeding” preterm newborns might be linked to obesity and cardiovascular complications later in life.

In the UAE, a recent study indicated a prevalence of 6.3% of preterm births in Abu Dhabi, the country’s capital [42]. If hospitals within the country are using different growth standards, it would be challenging to transfer infants between facilities and implement proper care and treatment measures [43]. Inconsistencies in assessing size and growth status may hinder effective communication between healthcare facilities, leading to potential misinterpretations and misclassifications. This lack of standardization complicates the establishment of uniform protocols for treatment and nutritional interventions, impacting both in-hospital care and post-discharge plans. Therefore, there is an urgent need for harmonizing growth assessment tools used in the UAE by prospectively evaluating international growth charts. Findings of our study suggest that using the INTERGROWTH- 21st standards might lead to a reduction in the diagnosis of extra-uterine growth restriction (EUGR). Thus, it is essential for future studies to examine the difference in outcomes of infant classified as EUGR using different charts. It is recommended to invest in more research to establish which charts are more predictive of clinical outcome and should be used in the UAE. Then involving decision-makers to ensure that all clinics and hospitals are using the same growth chart. This would facilitate direct comparisons, have positive consequences on clinical practices, and uniform the diagnosis of infants and children. Moreover, systematic compilation of existing data and regular estimations of preterm birth prevalence, employing consistent methods, are essential for evaluating the national burdens associated with preterm births.

### Strengths and limitations

To our knowledge, this is the first study to compare INTERGROWTH- 21st and Fenton growth charts in the UAE. However, the design of this study was not prospective and therefore we relied on anthropometric measurements assessed by the healthcare team at the study site. It is recommended to use other study designs (prospective or clinical trials) in future studies to reduce bias in anthropometric measurements. Data in this study were collected from a single center which limits the external validity of the study. Future studies with a more representative sample of infants in the UAE are needed to generalize the results to the whole population.

## Conclusion

This study has shown a significant difference in growth pattern assessment using Fenton curve and INTERGROWTH- 21st growth charts. However, the question remains which standard has a higher sensitivity for adverse long-term outcomes among preterm infants. Therefore, future large prospective studies are warranted to further define the predictive ability of these growth charts in identifying postnatal growth failure. National charts might be needed to accurately assess and monitor the growth of preterm infants.

## Data Availability

The datasets used and/or analyzed during the current study are available from the corresponding author on reasonable request.

## Abbreviations

AGA: Appropriate for gestational age
EUGR: Extrauterine growth restriction
U5MR: Under-5 mortality rate
IQR: Inter-quartile range
IUGR: Intrauterine growth restriction
LGA: Large for gestational age
SD: Standard deviation
SGA: Small for gestational age
UAE: United Arab Emirates
UOS: University of Sharjah
